# Fluid Intelligence and Psychosocial Outcome: From Logical Problem Solving to Social Adaptation

**DOI:** 10.1371/journal.pone.0024858

**Published:** 2011-09-21

**Authors:** David Huepe, María Roca, Natalia Salas, Andrés Canales-Johnson, Álvaro A. Rivera-Rei, Leandro Zamorano, Aimée Concepción, Facundo Manes, Agustín Ibañez

**Affiliations:** 1 Cognitive Development Center, Universidad Diego Portales, Santiago, Chile; 2 Faculty of Education, Universidad Diego Portales, Santiago, Chile; 3 Doctoral Program in Education, Pontificia Universidad Católica de Chile, Santiago, Chile; 4 Laboratory of Experimental Psychology and Neuroscience (LPEN), Institute of Cognitive Neurology (INECO) and Institute of Neuroscience, Favaloro University, Buenos Aires, Argentina; 5 Laboratory of Cognitive and Social Neuroscience, Universidad Diego Portales, Santiago, Chile; 6 National Scientific and Technical Research Council (CONICET), Buenos Aires, Argentina; University of Granada, Spain

## Abstract

**Background:**

While fluid intelligence has proved to be central to executive functioning, logical reasoning and other frontal functions, the role of this ability in psychosocial adaptation has not been well characterized.

**Methodology/Principal Findings:**

A random-probabilistic sample of 2370 secondary school students completed measures of fluid intelligence (Raven's Progressive Matrices, RPM) and several measures of psychological adaptation: bullying (Delaware Bullying Questionnaire), domestic abuse of adolescents (Conflict Tactic Scale), drug intake (ONUDD), self-esteem (Rosenberg's Self Esteem Scale) and the Perceived Mental Health Scale (Spanish adaptation).

Lower fluid intelligence scores were associated with physical violence, both in the role of victim and victimizer. Drug intake, especially cannabis, cocaine and inhalants and lower self-esteem were also associated with lower fluid intelligence. Finally, scores on the perceived mental health assessment were better when fluid intelligence scores were higher.

**Conclusions/Significance:**

Our results show evidence of a strong association between psychosocial adaptation and fluid intelligence, suggesting that the latter is not only central to executive functioning but also forms part of a more general capacity for adaptation to social contexts.

## Introduction

Fluid intelligence has been defined as the ability to think logically and solve problems in novel situations, independent of acquired knowledge [1]. Fluid intelligence reflects an individual's capacity for abstract thought and reasoning and it contrasts with so-called “crystallized intelligence” [2], which depends on previous knowledge and educational achievement. Undoubtedly, fluid intelligence is relevant to the process of analyzing novel problems, identifying patterns and relationships that underpin these problems and using logical extrapolation. Several tests have been proposed to measure this important function; the Raven Progressive Matrices (RPM) is the most widely-used task [3]. RPM is a psychometric non-verbal multiple choice test that evaluates the global index of intelligence. This index is traditionally inferred from a general factor of the underlying intelligence quotient (IQ) known as the *g* factor [3]. Although the RPM presents some caveats related to the fact that it is an extended and general measure, i.e., different processes seem to influence the RPM score, specifically, perceptual processing and analytic or analogical reasoning [4],those criticisms are expected for any general static cognitive measure. In fact, RPM is the most widely-used measure of g [5]. The *g* factor is thought to directly reflect a broad factor underlying several cognitive functions, such as observation and reasoning and, in this sense, evaluates a general intellectual capacity [6]. Usually, this measure is employed to evaluate perceptive skills and is recommended as a standard assessment in school populations [6]. During the application of the RPM, participants identify relevant information based on their perception of the spatial organization of an array of objects and their task consists of filling in the missing piece of a set of patterns. (See the Measures section for more details.)

Neuroanatomically, fluid intelligence has been related to frontal functioning [7]. Frontal lobe lesions have been found to affect performance on tests of fluid intelligence [7]–[9] and functional imaging studies that measure g have shown increased extensive activity in the frontal area of the brain [10]–[13].

Alongside the view that the frontal lobe constitutes the neural basis of fluid intelligence, frontal lobe functioning has also been linked to executive functioning and complex social behavior. Support for this comes from several lesion studies, which show that frontal lobe damage can alter behavior and social adaptation [14]–[22].

The relationship between fluid intelligence, novel problem solving and executive dysfunction has been extensively studied [7]–[11], [23]–[28] and the relationship between fluid intelligence and abstract reasoning [29]–[30] has already been established. However, research into the relationship between fluid intelligence and the behavioral domain is very scarce, especially as it pertains to psychosocial domains.

The term “psychosocial adaptation” refers to the quality of life in terms of social activities and relationships, sense of control and self-image. It includes multiple dimensions, such as social behavior, emotional regulation and the development of habits [31]. Given these characteristics, any approach that would be appropriate for assessing psychosocial adaptation would need to be both socially focused and broad enough to be able to capture an individual's subjective experience in several social domains, such as bullying, self- esteem, mental health and drug intake, among others.

Five social domains are particularly important for psychosocial adaptation: abuse of children and adolescents [32]–[36], bullying [37]–[41], drug use [32], [42]–[49], self-esteem [50]–[52] and mental health problems [50]–[58]. Moreover, the experience of abuse and bullying are positively associated with substance use and depression in children and adolescents [40], [59]–[63] as well as with negative effects on self-esteem [64]–[67]. To our knowledge, the relationship between said domains and fluid intelligence has not yet been studied.

Psychosocial adaptation is diagnostically and prognostically connected to neurological diseases [68], brain injury [69], aging, dementia and old age psychiatry [70]–[72], mental illness [73]–[76], ADHD and child/adolescent psychiatry [77]–[81], bipolar disorder and depression [82], schizophrenia [83]–[86] and epilepsy [87]–[88], to cite some examples. Neuropsychological and neurological assessments dominate the current neuropsychological and neuropsychiatric literature, but psychosocial considerations, in both the normal and the psychiatric/neurological population, are important. Psychosocial functioning represents an ecological evaluation of everyday adaptation and cognition, interlinked with cognition and emotion [89].

To our knowledge, no study with a large sample size, i.e., a random-probabilistic sample, has previously assessed the relationship between fluid intelligence and multiple measures of psychosocial adaptation. In the present study, we aimed to analyze this relationship by recruiting 2370 participants (controlled for educational level) who were tested on fluid intelligence using Raven's Progressive Matrices (RPM) and psychosocial outcomes, including measures of child abuse antecedents, bullying, self-esteem, mental health and drug use. In brief, we found that the lower the RPM scores were, the lower the level of psychosocial adaptation. Our results provide evidence of a strong association between fluid intelligence and psychosocial adaptation, suggesting that fluid intelligence is not only related to executive functions but is also a central component of the ability to adapt to social contexts.

## Materials and Methods

### Participants

This study was part of a Chilean regional county survey conducted during 2010 and designed to evaluate the psychosocial factors associated with academic achievement in scholars from socially vulnerable contexts. Only students belonging to public schools of Santiago de Chile were evaluated and all of the county's 21 public schools were included. Participants were recruited from primary schools, in the UK system, preparatory school covering the ages of 11–14 years old. From a total of 21 schools, 2370 students (age = 11.9 years, *S.D.* = 1.33; Sex = 46.8% female) were recruited using a random-probabilistic sample(maximum variance of 95% confidence with ±5% sample error). The students' ages ranged from 10–14 years, with the majority in the range of 11–13 years (Table 1). The participants came from socially vulnerable contexts. As expected for this population, the parents of the participants presented lower educational levels (75% completed only primary or secondary studies, without technical or university instruction). All schools that participated in this study approved the research. All participants and their parents or legal guardians gave signed, voluntary consent following the Declaration of Helsinki. This study was approved by the ethics committee of the Universidad Diego Portales – Santiago de Chile.

**Table 1:** Population and random sampling for the countries diagnosed.

	Academic Grade				
	5^th^	6^th^	7^th^	8^th^	Total
*N*	924	975	871	940	3829
*n*	573	657	565	575	2370

*n* = Sample achieved, *N* = Population. The table grade range (5 to 8th) represents the Chilean academic curricula including student of eight- to thirteen-year olds approximately.

### Measures

#### Fluid intelligence

The Standard Progressive Matrices version of the RPM was used as a measure of general intelligence, or *g* factor [3]. We used a standardized version for the sample under assessment [6]. The RPM included 60 spatial tasks divided into five blocks of 12 trials, from easiest to hardest. In each trial, participants were asked to complete a series of drawings by identifying the relevant features based on the spatial organization of an array of objects and choosing one object that matched one or more of the identified features.

#### Psychosocial adaptation

To assess bullying behavior, the Delaware Bullying Questionnaire [90] was used, which included two sub-scales of violent behavior. One sub-scale assessed the respondent as victimizer (*α = *.75); it contained questions such as “Within this year, how often have you done these kind of things at school:” “been part of a group that mocked a classmate who was alone”; “been part of a group that started a fight with another group”; “been part of a group that attacked members of another group”; “been part of a group that physically attacked a classmate who is alone”, among others. Another sub-scale assessed the respondent as victim (*α* = .72). It contained questions such as “have you been molested, while alone, by a group from your school”; “have you been physically attacked, while alone, by a group from your school”; “have you been in a group that has been attacked by another group”, among others. Answer options included “never”, “once”, “twice”, “three or four times” and “five times or more”. Both scales measured the event using the following options: it did not happen (1); it happened once during the year (2); it happened more than once during the year (3).

Domestic abuse of adolescents was assessed using the Conflict Tactic Scale (CTS) [91]. The subscales of psychological violence (*α* = .80) contained statements such as “has stopped talking to you for several days”; “has told you he/she didn't love you”; “has mocked you in front of other people”, among others. The questions assessing moderate physical violence (*α* = .84) included, among others, “has thrown things at you”; “has pulled your hair or ears”; “has pushed or shaken you”. Intense physical violence (α = .89) was assessed using statements such as “has given you a beating”; “has kicked, bitten or has given you a punch”; “has burned you with something (cigar, iron or hot water)”. The time scale included the following response options: “every day or almost every day”; “more than twice a week”; “more than twice a month”; “less than twice a month”; “has not happened in the last year, but it happened as a child” and “never”. Then, monthly and annual prevalence were calculated.

To assess drug intake, we used an international standardized scale [46] that evaluated monthly and annual prevalence of drug intake, specifically cannabis, cocaine, inhalants and non-prescribed stimulants (methylphenidate and methamphetamines).

Self-esteem was assessed using the Rosenberg's Self-Esteem Scale (*α* = .86) [92]. This scale includes statements such as “In general, I am happy with myself”; “Sometimes I feel like I am good at nothing”; “I feel like I have some good qualities”. Each item was measured on a scale ranging from “totally agree”, “agree”, “disagree”, to “totally disagree”. Rosenberg's Self-Esteem Scale ranks respondents on a scale between 0 (low self-esteem) and 20 (high self-esteem).

Mental health problems were assessed using a Spanish adaptation of the Perceived Mental Health Scale [93]. The Perceived Mental Health Scale includes questions such as “In the last four weeks, have you felt sad?”; “In the last four weeks, have you had attitude problems at school?”; “In the last four weeks, have you felt tired all the time?” The Mental Health Scale ranks respondents on a scale between 0 (no mental health problems) and 20 (maximum mental health problems).

#### Procedure

The assessments were performed by a team of trained social psychologists (*n* = 4) in each educational institution. In order to avoid cheating, the students were divided into two to four classrooms and the overall process of RPM and psychosocial test application was carefully supervised by the team of psychologists. The average duration of the assessment was 29 minutes (*S.D.* = 9.0) for the RPM and around 20 minutes for the psychosocial measures. There was a deadline of 45 minutes for the RPM and 30 for the psychosocial scale assessment. Only participants who finished all assessments within the time interval provided were included in the current study. Assessments were carried out at twenty-one institutions over a period of one month. The children's parents were notified about the procedure by the authorities of the educational institutions.

#### Statistical Analysis

The data were analyzed using SPSS software (Statistical Package for the Social Sciences, version 17.0). To assess the association between RPM scores and each measure of violence, correspondence analysis (CA) was used [94]. CA is a descriptive measure to represent contingency tables, i.e., tables in which the frequency of two or more qualitative variables are collected from a group of elements. CA allows the representation of the interdependence among variables measured using a nominal scale. This technique transforms non-metric data (ordinal and categorical variables) into metric data, allowing one-dimensional reduction (as a factorial analysis) and perceptual mapping (as a multidimensional analysis). In addition, ANOVA and χ^2^ were used as tests of independence. For the χ^2^ correlations, Cramer's *V* was computed. Cramer's *V* ranges between 0 and 1 to indicate the strength of association between two variables. For pairwise comparisons, Tukey's HSD *post hoc* tests were performed. To determine the relevance of the relationships, measures of the effect size *w* (for proportions) and *d* (for mean differences) were calculated [95]. The calculation of effect sizes allows the assessment of the magnitude of relationships beyond the mere reporting of *p*-values, which only specify the existence of statistically significant relationships. The calculation of effect sizes should temper the concerns about finding significant results solely on the basis of a large sample size and help avoid treating every significant result equally. To control for confounding variables, logistic regressions were run between the RPM and the binary variables, including parental educational levels, as predictors. ANCOVA was used to achieve the same control in evaluating the relationship between RPM and our measures of mental health and self-esteem.

## Results

### RPM scores

Five levels of scoring for the RPM were constructed in order to relate fluid intelligence to psychosocial adaptation. The total RPM index for each of the percentiles 5, 10, 25, 50, 75, 90 and 95 for each age group were obtained. Based on those indexes five scores were obtained (Table 2).

**Table 2:** RPM's five level score relating fluid intelligence with psychosocial adaptation.

Score	Level of intelligence	Percentile
1	High superior	≥95
2	Moderate superior	≥75 and <95
3	Average	>25 and <75
4	Moderate inferior	>5 and ≤25
5	Low inferior	≤5

The scoring was based on previous standardized studies reported in Chile in a sample of 4258 students (Ivanovic et al. [6]). Using this parameter a relatively normal participant distribution was observed in our five score levels: Score 1: 5.3%; Score 2:19.4%; Score 3: 48.2%; Score 4:19.9; Score 5: 7.2%.

### RPM and Bullying

The Delaware Bullying Questionnaire showed that 1 out of 3 (30.3%) students reported having exhibited violent behavior in the last year; 18.2% of the participants reported more than two episodes of violent behavior against other students and 51.5% reported that they had never performed a physical assault on another student. When the relationship between these results and the RPM scores was analyzed, the CA revealed a significant effect (χ^2^ (8, *N* = 3692) = 109.62, *p*<.001). To facilitate the interpretation of these data and following technical suggestions [92], table 3 shows the χ^2^ distances between the categories of each variable.

**Table 3:** RPM Scores associated to the bullying victimizer categories (χ^2^ distances).

	Score 1	Score 2	Score 3	Score 4	Score 5
Never	5.25	8.58	1.72	−18.98	−9.60
Once in a year	−3.47	−1.16	−0.27	4.80	1.35
Twice or more during the year	−2.22	−12.96	−2.45	21.09	14.31

Positive and higher values of χ^2^ distances are indicative of and strong association. Negative score are indicative of a lack of association.

**Figure 1:**
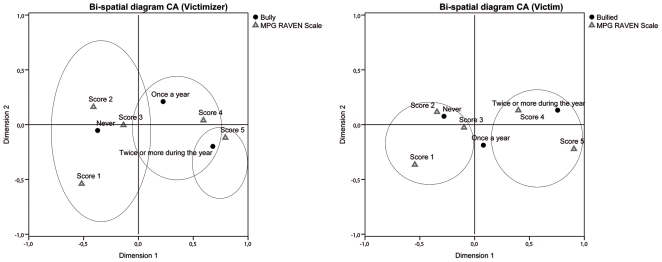
Bi-spatial diagram showing the relation between bullying behavior and SMP score (victimizer and victim). Circles display groups of categories close together.

Reduced or absent bullying behavior was associated with higher RPM scores. On the contrary, repetitive bullying behavior was related to lower RPM scores. The bi-space diagram shows the association between RPM scores and bullying behavior (Figure 1).

Regarding victimization, similarly to the victimizer scales, 1 out of 3 (30.1%) students reported having been exposed to violent behavior in the last year; 16% of the participants were exposed to more than two episodes of violent behavior against them; 52% reported to have never been exposed to physical assault from other students. When the relationship between victimization and RPM scores was analyzed, the CA revealed a significant effect (χ^2^ (8, *N* = 3704) = 67.03, *p*<.001). As before, Table 4 shows the χ^2^ distances between the categories of each variable and Figure 1 the bi-spatial diagram.

**Table 4:** RPM Scores associated to the bullying victim categories (χ^2^ distances).

	Score 1	Score 2	Score 3	Score 4	Score 5
Never	2.02	4.11	0.43	−3.72	−9.85
Once in a year	−0.00	−1.03	0.01	0.03	1.39
Twice or more during the year	−6.36	−5.27	−1.85	10.93	16.89

Positive and higher values of χ^2^ distances are indicative of and strong association. Negative score are indicative of a lack of association.

Similar to the results of the victimizer scale, a high correspondence was observed between reports of being a victim of violence more than once a year and lower RPM scores. At the same time, higher RPM scores seemed to be a protective factor against other's aggression.

### RPM and drug intake

**Figure 2:**
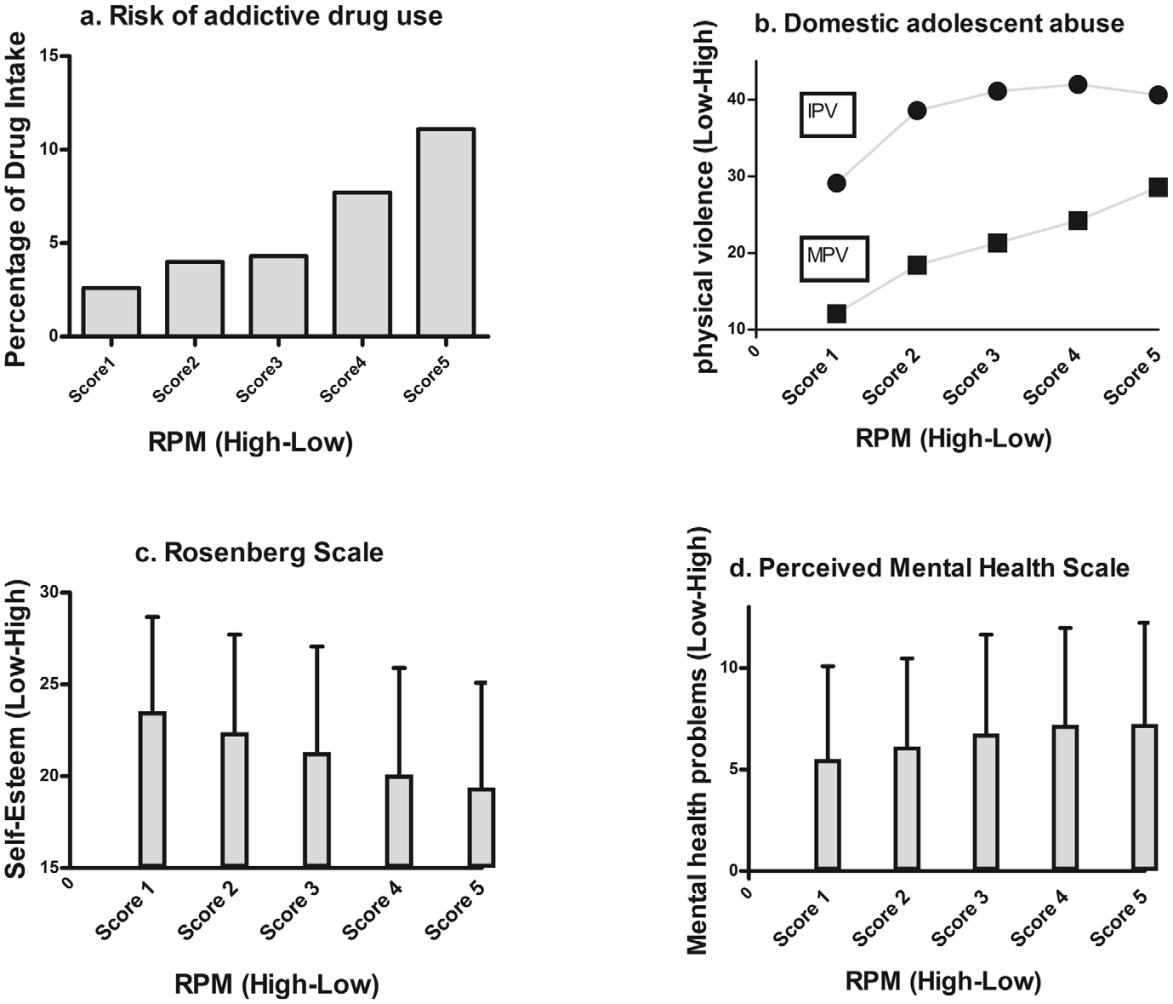
The relation between RPM and psychosocial outcome (drug intake, violence, self-esteem and mental health). (a) RPM scores and annual prevalence of drug intake (%). (b) RPM scores and annual prevalence's percentages of moderate (MPV) and intense physical violence (IPV). (C) RPM scores and self-esteem (Means and *S.D*.). RPM score and perceived mental health (Means and *S.D.*). In each subfigure (a, b, c, d), RPM measures of fluid intelligence are presented from left (Higher levels of intelligence) to right (Lower levels of intelligence).

Cannabis consumption showed an annual prevalence of 3%, followed by coca paste (2.2%) and cocaine (2%). The annual prevalence of use of all cocaine-related drugs, i.e., cocaine, coca paste and crack, was 3.3%; followed by inhalants (2.7%) and non-prescribed stimulants (2.2%). The composite score of drug intake presented an annual prevalence of 5.5%. This composite measure of drug consumption showed a significant association with RPM scores (χ^2^ (4, *N* = 3734) = 36.48, *p*<.001, *V* = .10) and a medium effect size (Cohen's *w* = .42). Lower scores of RPM were associated with higher percentages of drug use. Figure 2a shows the percentages for each RPM score.

### RPM and abuse of adolescents

The adolescent abuse scale showed a high annual prevalence of within-family psychological violence: 52.5%, 1 out of 2 participants had been a victim of family violence. The annual prevalence of moderate physical violence was 40.2% and the prevalence of intense physical violence was 21.4%. RPM scores were highly associated with the annual prevalence of moderate physical violence (χ^2^ (4, *N* = 3735) = 12.80, *p* = .012, *V* = .06, Cohen's *w* = .07 small effect size) and intense physical violence (χ^2^ (4, *N* = 3735) = 25.50, *p*<.001, *V* = .08; Cohen's *w* = .16, small effect size; see Figure 2b). Nevertheless, no association between psychological violence and RPM scores (4, *N* = 3735) = 4.48, *p* = 0.35, *ns*) was found.

### RPM and self-esteem

The self-esteem scale yielded an average score of 21.25 (*S.D.* = 5.74; range between 0-lower and 30-higher). A one-way ANOVA with the 5 RPM scores as a within-subjects factor revealed a strong effect (*F*(4, 3711) = 30.75, *p*<.001). *Post hoc* comparisons (Tukey's HSD test) show that in participants with lower RPM scores, lower reports of self-esteem were observed (Figure 2c). All post hoc comparisons were statistically significant at *p*<.001, except score 1 vs. score 2 and score 4 vs. score 5, which were not significant. The effect sizes of significant comparisons ranged from small to large (Cohen's *d* range = .20 to .77).

### RPM and perceived mental health

The average total score for mental health-related problems reported was 6.88 (*S.D.* = 4.76; range between 0-lower and 20-higher mental health-related problems). A one-way ANOVA with the 5 RPM scores as a within-subjects factor yielded a significant effect (*F*(4, 3699) = 8.67, *p*<.001). Similar to the results for the self-esteem measures, an inverse linear-like relationship was observed: the lower the RPM score, the higher the number of health problems reported (Figure 2d). Except for the pairwise comparison between scores 1–2, score 3–4 and score 4–5, which were not significant, all other comparisons yielded significant effects (Tukey's HSD Test, *p*<.001). The effect sizes of the significant comparisons were small (Cohen's *d* range = .14 to .36).

### RPM effects controlling for parental education

As noted above, we controlled for two possible effects that could weaken or even cancel out our results: socioeconomic status and parental educational level. The first was fixed within the design because all of the population came from the same socioeconomic group and had a socioeconomic status of middle-low and lower. Despite this, the intelligence scores varied and showed a normal distribution, independent of socioeconomic status, which in this study was a constant. Therefore, the effects can be seen as being independent of this condition. Parental educational level was measured in terms of years of study (0 to 8 points) and was covaried with each of the relationships that were tested above. For the association between the RPM score and the measurement of bullying as the perpetrator, “Bully”, a logistic regression was used, with the following predictors: RPM score, father's educational level and mother's educational level. ‘Bully’ was coded as a dependent variable as 0 = Never, with a prevalence of 48.5% and 1 = Once a year or twice or more during the year, with a prevalence of 51.5%).RPM had an important effect in the expected direction (low RPM score –lower IQ– greater chance to trigger bullying, Table 5). We also found a similar outcome with ‘Bullied’ as the dependent variable. (This was coded 0 = Never, with a prevalence of 52% and 1 = Once a year or twice or more during the year, with a prevalence of 48%.) In the case of “Bullied” only RPM was statistically significant in the predicted direction (Table 5).A similar finding was observed for the variable ‘use of illicit drugs’, annual prevalence (0 = Never, 1 = Yes, Table 5); and ‘intense physical violence’ (Table 5). For “moderate physical violence”, all effects were significant (Table 5). However, the most relevant outcome is that the RPM effects described remain significant over and above the influence of the parental educational levels. Finally, for the associations between RPM with self-esteem and perceived mental health, we used ANCOVAs. In both cases, the effects found for RPM were retained. For self-esteem *F*(4, 3531) = 28.59, *p*<.001. The effects of the parental educational levels were not significant. For mental health, *F*(4, 3520) = 8.91, *p*<.001. Again, the effects of the parental educational levels were not significant.

**Table 5:** Results from logistic regressions with RPM, father education, and mother education as predictors.

	RPM			Father's education level			Mother's education level		
Regressand	*β*	Wald	OR CI_95%_	*β*	Wald	OR CI_95%_	*β*	Wald	OR CI_95%_
Bully	.33**	76.37	1.29–1.50	.030*	4.47	1.00–1.06	−.014	.91	.96–1.01
Bullied	.22**	36.76	1.16–1.34	.015	1.12	.99–1.04	−.018	1.62	.96–1.01
Use of illicit drugs	.43**	28.52	1.31–1.79	−.006	.04	.94–1.06	−.009	.09	.93–1.05
Intense physical violence	.25**	31.98	1.18–1.40	−.013	.60	.95–1.02	.014	.64	.98–1.05
Moderate physical violence	.09*	6.10	1.02–1.18	−.031*	4.59	.94–.99	.044**	9.40	1.02–1.08

*p<.05;

**p<.01.

## Discussion

This is the first large sample study to assess the relationship between fluid intelligence and psychosocial adaptation. The overall results of our study suggest that fluid intelligence has a strong association with psychosocial measures. We found a linear relationship between RPM and measures of physical violence as the victimizer: the lower the RPM, the higher the violence score. We found a similar relationship between RPM and measures of violence pertaining to the victim role. No relation between RPM and psychological violence was found. Drug intake (especially for cannabis, cocaine and inhalants) was higher when RPM was lower. Self-esteem reports were modulated by 5 levels of RPM (from lower to higher) in a simple way: the lower the RPM, the lower the self-esteem. Finally, similar results were found for the mental health measurement: higher rates of health were observed when RPM was higher. All effects remained even when covaried with parental educational level.

Deficits in fluid intelligence, executive functioning and social adaptation have been described after frontal lobe lesions [14]–[18], [20]–[22]. Because all of these deficits have been linked to a common brain area, it is important to investigate the relationship between these deficits. While the relationship between executive functioning and fluid intelligence has already been established, investigations assessing the relationship between the latter and social adaptation are limited. Our data indicate that fluid intelligence is associated not only with executive function but is also a relevant component of psychosocial adaptation.

Complex modern societies demand a strong capacity for social adaptation. Bullying and violence, addictive behavior, perceived mental health and self-esteem are strongly linked with quality of life [96]–[102]. Our data evidence a straightforward association between levels of fluid intelligence and the degree of social adaptation. This is a novel result and opens up a new branch of research relating “cold” measures of intelligence to “hot” measures of socially-dependent behavior. Nevertheless, literature from other domains has produced some evidence of this relationship. For instance, Perry et al. [102] reported predictors of outcome in 332 children, aged 2–7 years, enrolled in the community-based Intensive Behavioral Intervention (IBI) program in Ontario, Canada and found that in the subset of children who had an IQ score available at intake (n = 151), there were significant and strong correlations between initial IQ and all outcome variables, mainly scales of adaptive behavior. Psychological adaptation problems, e.g., attention deficits, violence, patterns of antisocial, impulsive, norm-violating, sensation seeking and externalizing tendencies and substance use, have been linked to behavioral disinhibition [103]. In turn, behavioral disinhibition has been associated with reduced working memory and short-term memory capacity, as well as with lower IQ [104]. Various studies have shown an association between IQ in childhood or early adulthood and mortality in later life [105]–[109]. Consistent with our results, cognitive research has shown that sensitive parenting is linked with higher child IQ [110]. On the other hand, children who witness domestic violence tend to have significantly lower IQs [111] than their non-exposed peers [112]–[113]. There is also consistent evidence that relates low intelligence and delinquency [114]–[117]. As an example, Koolhof et al. [114] found that delinquents with low IQ were more behaviorally and cognitively impulsive than higher IQ delinquents. Additionally, low IQ offenders exhibited greater deficiency in empathy and guilt feelings that those with high IQ. Impulsivity, therefore, appears to be a key characteristic of low IQ.

Even though the aforementioned studies suggest a link between IQ and behavioral outcomes, no previous study has directly assessed the association between fluid intelligence and psychosocial adaptation. Ours is the first study to look for this association using a larger cohort and several measures of psychological adaptation. In addition, our sample is random and probabilistic, which is an uncommon design in studies of its type, they are usually carried out using convenience sample or intentional samples and provides greater generalizability of results and greater statistical power.

Our results need to be extended and replicated along several dimensions. First, our sample represents a socially vulnerable population and further studies should asses the relationship between fluid intelligence and psychosocial adaptation in other socioeconomic groups. In addition, further studies should include not only measures of fluid intelligence but also measures of crystallized intelligence to compare the effects of educational and cultural experience in interaction with fluid intelligence. Also, the inclusion of more objective and quantitative measures of psychosocial adaptation, such as experimental designs or brain studies, would provide an interesting, if challenging, approach to study the relationship between social adaptation and fluid intelligence.

Does low fluid intelligence itself make a person more vulnerable to social adaptation problems? Or is it that fluid intelligence is correlated with the situation in which a person lives? For example, certainly lower intelligence is correlated with lower family income, so are we seeing the effects of poverty? Unfortunately, this is impossible to answer these questions with our data and further research is needed to address this topic. Nevertheless, at least some data suggest that the range of fluid intelligence is to some extent independent of socioeconomic and educational levels. Because we found that the effects of fluid intelligence on all measures of psychosocial adaptation remain once both socioeconomic and educational levels are covaried, we speculate that fluid intelligence is not completely dependent on socio-education. The association between fluid intelligence and psychosocial adaptation is very consistent in our data, the higher the first, the higher the second, independent of socioeconomic status and socio-educational level, which was tested with statistical techniques for covariance.

A second issue concerning our results is the possible relationship between high fluid intelligence and social desirability. Specifically, could it be that fluid intelligence affects the tendency to answer questions in a socially desirable way? Unfortunately, we don't have any direct way to address this point and the possibility that social desirability could act as a moderator effect in children with high RPM scores cannot be discarded. However, the relationship between a high RPM score and better psychosocial adaptation could be interpreted, from our point of view, as suggesting that children with higher fluid intelligence exhibit more adaptive behaviors than those who show low scores. Fundamentally, this can be sustained in students who report being less frequent victims of bullying at school, which in turn also correlates with a lower prevalence of domestic violence. Precisely, greater fluid intelligence implies that subjects will use the most effective strategies to deal with becoming a victim of aggression, both in school and at home. Therefore, the fact that children show higher fluid intelligence shouldn't necessarily mean they avoid giving a sincere response about their situation. Future studies assessing implicit, not only explicit, measures of psychosocial adaptation will help to clarify the possible role of the moderator effect.

Finally, the relationship between executive function (EF), psychosocial adaptation and fluid intelligence calls for research. It is known that EFs are comprised of self-monitoring abilities. These functions are essential to goal-directed behavior, allowing us to maintain, update and integrate information to adapt and move within our environment [118].

### Conclusion

This is the first report suggesting a clear relationship between fluid intelligence and psychosocial adaptation evaluated in several domains. These results call for a new branch of research that combines a neurocognitive approach to fluid intelligence with study of psychosocial adaptation.
